# Supplementary material for: Prevalence of undernutrition and associated factors among adults taking antiretroviral therapy in sub-Saharan Africa: A systematic review and meta-analysis
Source: PLoS One. 2023 Mar 24;18(3):e0283502. doi: 10.1371/journal.pone.0283502 (PMC10038308; doi:10.1371/journal.pone.0283502)
Supplement: S3 Table — (DOCX) [file pone.0283502.s003.docx]

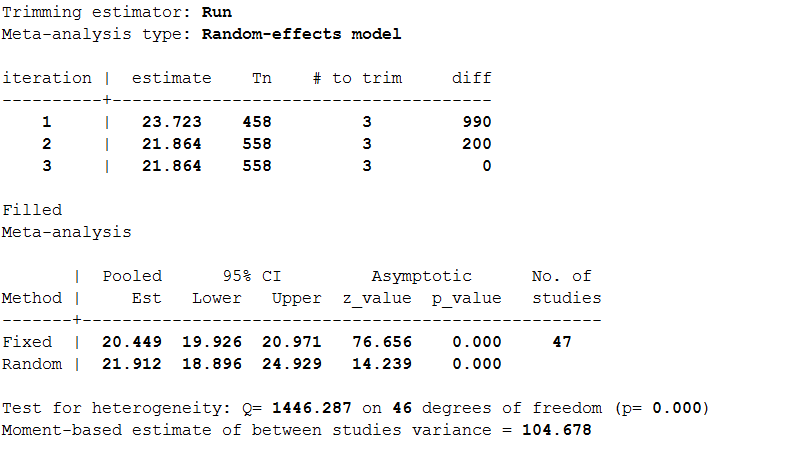


**S3 Table. Trim and fill analysis of the prevalence of undernutrition and associated factors among adult PLWHIV in SSA, 2009-2021**
