# Supplementary material for: Prevalence of undernutrition and associated factors among adults taking antiretroviral therapy in sub-Saharan Africa: A systematic review and meta-analysis
Source: PLoS One. 2023 Mar 24;18(3):e0283502. doi: 10.1371/journal.pone.0283502 (PMC10038308; doi:10.1371/journal.pone.0283502)
Supplement: Attachment — Submitted filename: SUmmary of comments.docx [file pone.0283502.s004.docx]

**Prevalence and associated factors of under-nutrition among adults taking anti-retroviral therapy in sub-Saharan Africa: A systematic review and meta-analysis**

1. **General comment:** Relevant topic, consolidating evidence about the relationship between under-nutrition and ART.

However, the whole article needs to be revisited to make sure that English language is well written especially writing the article in a past tense. The authors should define abbreviations used for the first time

2. **Abstract:**

**Background**: Lacks the research gap and the broad aim/objective of conducting the study. **Method**: Some sentences written in present tense instead of past tense.

3. **Main body:**

**Introduction**: Revisit the first sentence in the first paragraph (Probably the authors meant that the rate of new HIV infection is decreasing Worldwide). 5^th^ and 6^th^ paragraphs talked about previous studies which also determined prevalence of under-nutrition and associated factors with HIV. However, the authors did not show the gap intended to be filled by the current study. It is not clear why the current meta-analysis was done as there were already some meta-analysis done as indicated in the literature review. However, the rationale is well stated.

**Method**: Include a table showing the PICO of the systematic review. Search words shown are only those for PubMed. I suggest you list all the search words considered in every search engine without giving the details of how the different search words were related (OR/AND). Give initials for the third author.

**Results**: Arrange the table and figures e.g. the PRISMA as they are discussed in the results section.

**Discussion**: Avoid repeating results in the discussion section
