# Supplementary material for: Prevalence of undernutrition and associated factors among adults taking antiretroviral therapy in sub-Saharan Africa: A systematic review and meta-analysis
Source: PLoS One. 2023 Mar 24;18(3):e0283502. doi: 10.1371/journal.pone.0283502 (PMC10038308; doi:10.1371/journal.pone.0283502)
Supplement: Attachment — Submitted filename: Nutrition.docx [file pone.0283502.s005.docx]

Prevalence and associated factors of under-nutrition among adults taking anti-retroviral

therapy in sub-Saharan Africa: A systematic review and meta-analysis

This was a systematic review to estimate pooled prevalence of under nutrition and associated factors among PLHIV in sSA.

Comment:

Overall:

Manuscript need English proof reading. Several areas past tense have not been used, conjunction missing. Message not clearly communicated

Title: Consider revising it to “Prevalence of under-nutrition and its associated factors among adults taking anti-retroviral therapy in sub-Saharan Africa: A systematic review and meta-analysis”

Define HIV appropriately as Human immunodeficiency virus. Do dot prefer people as HIV infected, you may consider using people living with HIV as the former is regarded as stigmatizing

Introduction:

There are about 7 paragraphs. Not clearly aligned. Consider reducing number of paragraphs to about 4 and each should carry specific message. Stating bidirectional relationship between ART and undernutrition without describing it not enough.

22 times more likely, if the study did not use risk ration, interpretation is not right.

Methods:

Needs English proof reading

Issues such as ‘The review is aimed……’; ‘databases are accessed…..’ needs revision to past tense. The word on the other had was used severally in the manuscript but not showing clear contrast

‘…..to find associated factors of under-nutrition’ could be factors associated with under-nutrition

Results.

There is unnecessary bolding of numbers or reference (eg **figure xxx**) , a total of **22316 etc**

‘Sensitivity analysis is performed…..’ Needs to be past tense through out the manuscript

Odds ratios were not appropriately interpreted. The odds of X is 2 times that of Y. Not 2 times higher

‘……1.94 times more likely ….’ Is not appropriate, can be written 1.94 higher odds of having under-nutrition. This is not a risk ratio, you can’t say more likely

Discussion:

Needs revision

Like introduction, consider few paragraph with specific message

1- Paragraph- summarize main findings
   2-3 Paragraphs- consider main factors to expand

4- para- consider limitations and strength

5- conclusion
