# Supplementary material for: Prevalence of undernutrition and associated factors among adults taking antiretroviral therapy in sub-Saharan Africa: A systematic review and meta-analysis
Source: PLoS One. 2023 Mar 24;18(3):e0283502. doi: 10.1371/journal.pone.0283502 (PMC10038308; doi:10.1371/journal.pone.0283502)
Supplement: Attachment — Submitted filename: Response to reviewers.docx [file pone.0283502.s006.docx]

**Response to academic editor and reviewers**

**Response to academic editor:**

| **Comments** | **Response** |
| --- | --- |
| Please ensure that your manuscript meets PLOS ONE's style requirements, including those for file naming. | We have made corrections to meet PLOS ONE's style requirements, including those for file naming. |
| Please clarify the sources of funding (financial or material support) for your study. List the grants or organizations that supported your study, including funding received from your institution. | As we did not receive any funding for this study, we included the statement “The authors received no specific funding for this work” within the manuscript. |
| We note that you have stated that you will provide repository information for your data at acceptance. Should your manuscript be accepted for publication, we will hold it until you provide the relevant accession numbers or DOIs necessary to access your data. If you wish to make changes to your Data Availability statement, please describe these changes in your cover letter and we will update your Data Availability statement to reflect the information you provide. | I cannot get the data availability statement in our manuscript which says “provide repository information for your data at acceptance”.  Data availability statement in our manuscript is written as “All relevant data are within the manuscript and supporting information files” |
| We note that you have referenced (ie. Bewick et al. [5]) which has currently not yet been accepted for publication. Please remove this from your References and amend this to state in the body of your manuscript: (ie “Bewick et al. [Unpublished]”) as detailed online in our guide for authors | Similarly, I cannot get a reference (i.e. Bewick et al. [5]) in the manuscript. |
| Please include captions for your Supporting Information files at the end of your manuscript, and update any in-text citations to match accordingly. | I made corrections regarding captions of tables, figures and supplementary materials as per the journal guideline. |

**Response to reviewer #1**

| **Comments** | **Response** |
| --- | --- |
| 1. **General comment:** the whole article needs to be revisited to make sure that English language is well written especially writing the article in a past tense. The authors should define abbreviations used for the first time. 2. The authors should define abbreviations used for the first time. | We noted several English proofreading problems in the manuscript. We have made corrections word by word. I also invited English language editors to minimize language problems. Grammatical errors are also corrected.  It is acceptable that abbreviations should be defined in the first use and we made corrections. |
| 3. **Abstract:**   **Background**: Lacks the research gap and the broad aim/objective of conducting the study.  **Method**: Some sentences written in present tense instead of past tense. | We incorporate the research gap and objective in the background section of the abstract.  We changed verbs to past tense in the method section. |
| 4. **Main body:**   **Introduction**: Revisit the first sentence in the first paragraph (Probably the authors meant that the rate of new HIV infection is decreasing Worldwide). 5^th^ and 6^th^ paragraphs talked about previous studies which also determined prevalence of under-nutrition and associated factors with HIV. However, the authors did not show the gap intended to be filled by the current study. It is not clear why the current meta-analysis was done as there were already some meta-analysis done as indicated in the literature review. However, the rationale is well stated. | The first and second paragraphs are merged and paraphrased. Meta-analysis was done in Ethiopia, not at the sub-Sahara Africa level. There are a number of studies but lacks consistency. Besides, we believe a single country figure will not represent the region. Therefore, this is the first review in sub-Sahara Africa and this statement is incorporated in the paragraph. |
| **Method**: Include a table showing the PICO of the systematic review. Search words shown are only those for PubMed. I suggest you list all the search words considered in every search engine without giving the details of how the different search words were related (OR/AND). Give initials for the third author. | Thank you for this comment and PICO table is incorporated and makes the study more clear.  Search words and Mesh terms were used only for PubMed because it is an open access database. We have no institutional access to Embase, Scopus and other databases which is described under limitation. Therefore, we didn’t use different search words for these databases. However, we searched articles in Google scholar which is comprehensive and include articles indexed in those databases. |
| **Results**: Arrange the table and figures e.g. the PRISMA as they are discussed in the results section. | We arranged and make results in line with the PRISMA chart. |
| **Discussion**: Avoid repeating results in the discussion section | We noted some repetition of results in the discussion section and removed it. |

**Response to reviewer #2**

| **Comments** | **Response** |
| --- | --- |
| **Overall comment:**  Manuscript need English proof reading. Several areas past tense have not been used, conjunction missing. Message not clearly communicated  Title: Consider revising it to “Prevalence of under-nutrition and its associated factors among adults taking anti-retroviral therapy in sub-Saharan Africa: A systematic review and meta-analysis”  Define HIV appropriately as Human immunodeficiency virus. Do dot prefer people as HIV infected, you may consider using people living with HIV as the former is regarded as stigmatizing | We admit the manuscript had language problems and we undergo thorough revision and language editors were also invited to help us communicate the message clearly.  **Title**. It sounds more logical if the title is rephrased and done as indicated by the reviewer.  The abbreviation HIV is defined appropriately. We also admit the comment of stigmatizing expression of “HIV infected…” We change this expression throughout the manuscript. |
| Introduction:  There are about 7 paragraphs. Not clearly aligned. Consider reducing number of paragraphs to about 4 and each should carry specific message. Stating bidirectional relationship between ART and under-nutrition without describing it not enough.  22 times more likely, if the study did not use risk ration, interpretation is not right. | The paragraphs in the introduction are abridged to four and each carries specific messages. Regarding the interpretation of the odds ratio, we agree it is preferable to interpret as indicated and we modify throughout the manuscript. |
| Methods:  Needs English proof reading | We changed the tenses in methodology and other sections as commented. |
| There is unnecessary bolding of numbers or reference (e.g. **figure xxx**) , a total of **22316 etc** | Unnecessary bolding of words or numbers are avoided. We tried to correct the grammatical errors made throughout the manuscript including in the result section. |
| Discussion:  Needs revision  Like introduction, consider few paragraph with specific message   1- Paragraph- summarize main findings    2-3 Paragraphs- consider main factors to expand   4 - para- consider limitations and strength  5 - Conclusion | In the discussion section, we also merge paragraphs with similar messages. Even though there are few studies that limit the discussion, we tried to add other interpretation guides. |
