# Supplementary material for: Prevalence of undernutrition and associated factors among adults taking antiretroviral therapy in sub-Saharan Africa: A systematic review and meta-analysis
Source: PLoS One. 2023 Mar 24;18(3):e0283502. doi: 10.1371/journal.pone.0283502 (PMC10038308; doi:10.1371/journal.pone.0283502)
Supplement: Attachment — Submitted filename: Comments.docx [file pone.0283502.s007.docx]

Prevalence of under-nutrition and associated factors among adults taking antiretroviral

therapy in sub-Saharan Africa: A systematic review and meta-analysis

**Introduction**:

- Third sentence, first paragraph: Look for more than one proof for food insecurity in Africa or better look for a systematic review
- Make sure you define all the abbreviation when used for the first time e.g. ART
- Second paragraph: The bi-directionality was not shown; you only showed that malnutrition worsens HIV. The effect of HIV on malnutrition is missing
