# Supplementary material for: Prevalence of undernutrition and associated factors among adults taking antiretroviral therapy in sub-Saharan Africa: A systematic review and meta-analysis
Source: PLoS One. 2023 Mar 24;18(3):e0283502. doi: 10.1371/journal.pone.0283502 (PMC10038308; doi:10.1371/journal.pone.0283502)
Supplement: Attachment — Submitted filename: Response to reviewers #2.docx [file pone.0283502.s008.docx]

**Response to reviewer #1**

**Comment**: Third sentence, first paragraph: Look for more than one proof for food insecurity in Africa or better look for a systematic review

**Response:** I have included additional systematic review and primary studies to strengthen the evidence.

**Comment**: Make sure you define all the abbreviation when used for the first time e.g. ART

**Response**: Abbreviations are defined in the first usage throughout the manuscript.

**Comment**: Second paragraph: The bi-directionality was not shown; you only showed that malnutrition worsens HIV. The effect of HIV on malnutrition is missing.

**Response**: The effect of HIV on malnutrition is presented in detail.

**Response to reviewer # 3**

**Comment:** The interpretation of this review has a bias towards comparing outcomes for Ethiopia with other published outcomes about Ethiopia. Given that all the authors are from Ethiopia, perhaps the authors want to reframe this study title and objectives to having a particular focus on Ethiopia.

**Response:** The use of systematic reviews and meta-analysis studies reported from Ethiopia for interpretation and comparison was due to a lack of similar or related studies in other countries, not the authors' origins in Ethiopia. We tried our best to search for meta-analysis results conducted elsewhere outside of Africa too.  Of course, we added one meta-analysis study conducted on a similar topic but carried out only among women for additional comparison and interpretation. Notably, 11 out of 44 studies were conducted outside of Ethiopia, and as long as studies are systematically searched and no eligible studies were left in the final analysis, the result can be taken as representative of sub-Sahara African region. In spite of that, we mentioned the dominancy of studies from Ethiopia under the “limitation of the study”.

**Comment**: Wherever point estimates are provided confidence intervals should be included – for both this study, and the supporting background text.

**Response:** It is accepted and we included confidence intervals for different point estimates presented in the manuscript.

**Comment:** Inclusion criteria –prospective studies were not included?

**Response**: All observational studies reporting prevalence, incidence, and/or determinants were searched. But we didn’t find studies with prospective cohort designs, and only one retrospective cohort study was included.

**Comment:** How was under-nutrition defined?

**Response**: Even though there is no single indicator of nutritional status in adults, a low body mass index (BMI) of < 18.5 kg/m2 or underweight is an indicator of advanced malnutrition. The risk becomes higher when patients have chronic diseases like HIV. If it is not prevented or early detected, it will lead to "wasting syndrome," a sign of an advanced immune-compromised state. It was also demonstrated that starting antiretroviral therapy (ART) accelerated mortality (6 months) if the patient's BMI was less than 18. An additional point regarding definition of under-nutrition is added in the manuscript. All of the studies used a BMI of < 18.5kg/m^2^ to define under-nutrition.

(Koethe, John R., and Douglas C. Heimburger. "Nutritional aspects of HIV-associated wasting in sub-Saharan Africa." *The American journal of clinical nutrition* 91.4 (2010): 1138S-1142S.

<https://emedicine.medscape.com/article/2058483-overview>

**Comment:** Newcastle Ottawa Scale is good but it is stated that “A score of greater or equal to 6 points was considered “good” and included in the study”. Summary scores are a misleading way to assess quality. Further, if this was done, the number of studies excluded because they were not considered “good” should be included in the flow diagram and results section narrative

**Response**: The Newcastle Ottawa Scale, JBI, and Cochrane risk of bias assessment are the commonly used tools to screen the quality of published studies. When using NOS, it is difficult to make a qualitative judgment for eligibility without a cut-off point. The domain of assessment uses the star method, which is rated out of 10 points for cross-sectional studies. There are also studies published in PLOS ONE using similar judgment. We agree that studies that were not considered "good" should be reported in the flowchart. However, no study is excluded from the analysis due to poor quality.

**Comment**: A funnel plot is noted in the abstract and results, but not in the methods.

**Response**: It is incorporated in the method section as indicated.

**Comment**: Subgroup and sensitivity analyses are described in the results, but not the methods.

**Response**: It is incorporated in method section too.

**Comment:** I^2^ is not the most appropriate test for heterogeneity when the outcome measure is a proportion. As such, it is unsurprising that I^2^ is >90%. (Tau^2^ would have been better).

Yes it is true that both Q test with p value and I^2^ are a relative measure of heterogeneity. They don’t tell us exactly the magnitude of variation of effect sizes among the studies. Therefore, high I^2^ in the context of proportional meta-analysis does not necessarily mean that data is inconsistent. As such, the results of this test should be interpreted conservatively. It is advised to use I^2^ as a criterion for a decision whether a subgroup analysis or moderato analysis is indicated.

On the other hand, τ^2^ or Tau^2^ is **an estimate of the between-study variance in a random-effects meta-analysis**. The square root of this number (i.e. tau) is the estimated standard deviation of underlying effects across studies. T^2^ is not used itself as a measure of heterogeneity but is used in two other ways: (1) it is used to compute Tau; and (2) it is used to assign weights to the studies in the meta-analysis under the random-effects model. Tau is used for computing the *prediction interval*. Tau is a useful first indication of the extent of this dispersion. However, the *prediction interval* is a more direct and more easily interpretable indicator.

Michael Borenstein et al. (2009), Introduction to Meta-Analysis, Chichester (UK): Wiley.

Therefore, in our meta-analysis study the following is incorporated.

Tau^2^ = 98.83, Tau = 9.94, prediction interval (13.8 – 33.68), 95% CI confidence interval, (20.69 – 26.79)

With high heterogeneity, prediction intervals will be wider than confidence intervals, and can be considered a more conservative way to incorporate uncertainty in the analysis. Where possible, it is suggested estimation of prediction intervals alongside with confidence intervals, especially for prevalence and incidence estimates and we include this concept.

*Barker, Timothy Hugh, et al. "Conducting proportional meta-analysis in different types of systematic reviews: a guide for synthesis of evidence." BMC Medical Research Methodology 21.1 (2021): 1-9.*

**Comment:** The search strategy is not highly sensitive (e.g. “Anti-Retroviral Therapy” is a very limited term) and this should be noted as a limitation. Preferably, standard search terms would have been used (e.g. see search terms used by Cochrane, or search strings used by other reviews cited by this review, e.g. ref 10).

**Response**: We believe that we have searched enough articles using sufficient key words. We were having difficulty finding alternative search terms for the key word "Anti-Retroviral Therapy". Even the suggested reference by the reviewer didn’t include this word. We accept that truncations were not used. Additionally, the Cochrane handbook for systematic review recommends to avoid using too many *different* search concepts but a wide variety of search terms. ([www.training.cochrane.org/handbook](https://training.cochrane.org/handbook)). However, we don’t mean that the recommendation for maximizing the number of alternative key words is not important in our future work. Regarding duplication of terms, the second search strategy connected by “AND” was used to access potential case-control and retrospective cohort studies that might only report the associated factors (determinants) without the prevalence or incidence of under-nutrition. We believe that no study is leftover other than based on eligibility criteria in our final meta-analysis and it may not be necessary to put the search strategy as a crude limitation.

**Comment**: Results “Regarding the publication year, 11 articles were published in 2020 followed by 9 articles in 2017. Besides, only one article was reported in 2010” This is an anecdotal way of describing year of publication, given that 44 studies were included. Please reconsider how to present these data.

**Response:** This result is rephrased in a more informative way. **T**he detail is displayed in table 2 and description is reserved only for the majority of studies and range of year of publication.

**Comment**: Tables need legends to explain the acronyms

**Response**: Legends are added in some tables to avoid confusions. For example “ART” in table 2.

**Comment**: Figure 3. This is a very unconventional forest plot that does not display pooled estimates and confidence intervals well.

**Response**: Figure 3 is a sensitivity analysis graph. It, like the funnel plot, serves as a visual guide to publication bias. The graph shows estimates are within the confidence interval limit and removal of studies to minimize publication bias is not required. The sensitivity analysis indirectly supports the risk of bias assessment, which was arbitrarily assessed using NOS.
