# Supplementary material for: Prevalence of undernutrition and associated factors among adults taking antiretroviral therapy in sub-Saharan Africa: A systematic review and meta-analysis
Source: PLoS One. 2023 Mar 24;18(3):e0283502. doi: 10.1371/journal.pone.0283502 (PMC10038308; doi:10.1371/journal.pone.0283502)
Supplement: Attachment — Submitted filename: Response to reviewers V_3.docx [file pone.0283502.s009.docx]

**Response to reviewers #4**

First, I would like to thank the reviewers for taking the time to provide us with invaluable comments that enrich the quality of the manuscript.

**Comment**: Title: it seems the review includes adults living with HIV, whatever their ART status, fig5 showing sub-analysis by ART status, I would therefore suggest changing the title to fit better with the review.

**Response**: Yes, the manuscript includes adults living with HIV irrespective of their ART status, and the pooled prevalence is reported in the document. However, subgroup analysis by different relevant characteristics is common in order to better understand the results by different categories. The sub-group analysis was made to look into whether taking ART has made a difference in nutritional status. There is evidence that taking ART improves nutritional status. Sub-group analysis is made for description, not inference. We believe sub-group analysis by different variables will not demand changing the title.

**Abstract:**

**Comment**- Undernutrition has several definitions (low BMI, low weight, small mid-upper arm circumference, micronutrients deficiencies), this needs to be defined clearly in the abstract, especially as the background talks also about food insecurity which can be confusing. There are many terms used (malnutrition, wasting etc..) in the abstract and throughout the paper, which needs to be harmonized

**Response**: Though malnutrition also includes overnutrition, in this study and commonly, malnutrition, undernutrition, underweight, wasting, and malnourishment are all used in the same context. All of the studies included in this meta-analysis defined malnutrition as low BMI (less than or equal to 18.5 kg/m2), as indicated in the main text. Food security was mentioned as one of the causes of malnutrition in SSA. However, as it is not the scope of this study, we removed it from the statement. We put the operational definition in the first statement in brackets.

**Comment**: Methods section needs to be a bit more detailed (inclusion criteria, study period, study design and data extracted, especially related to the associated factors)

**Response**: Accepted and we add some details in methods part.

**Comment**: Results section could start with the number of total records identified with the search strategy before to give the final number.

**Response**: Different articles present the results in different ways in their abstracts. Overall, we used the **PRISMA 2020 Abstracts Checklist** to structure our abstract. Because the details are described in text and using a flow chart in the main result section, we prefer not to present the number of total records here.

**Comment**: Given the heterogeneity of the selected studies, whether in terms of study design, population, context, year etc.., I would be extremely careful while comparing the prevalence by country or years. I would rather focus on the HIV-related associated factors, which can be the more useful to target to further improve the nutritional status of people living with HIV.

**Response**: Thank you for the concern. Sub-group analysis is used in meta-analysis to describe phenomena by different segments rather than for inference. It is also used to identify the source of heterogeneity. Inference is made based on the major objective of the study, i.e., the pooled estimate of prevalence and associated factors. Therefore, we removed the sub-group result from the abstract as it was not our major finding. Additionally, we avoid comparison statements like "the highest" for the sub-group results presented in the main text.

**Introduction: overall this part lacks a bit of structure and justification**

**Comment**: First paragraph should explain the HIV epidemic among adults, and challenges of HIV care, especially in sub-Saharan Africa, I don’t think the part about food insecurity should be there.

**Response**: We really appreciate the comment on the structural organization of the contents presented in the introduction. A number of literatures on food insecurity were added as per previous reviewers’ comments on why the "sub-Saharan Africa region" was selected as the study area. It was to show the high burden of food insecurity and HIV transmission in SSA, which puts these patients at high risk for malnutrition. However, we abridged the statements on food insecurity and brought them to the third paragraph, where the evidence gaps are presented. Similarly, we added some statements describing the epidemiology of HIV among adults and the challenges of HIV care in SSA as background statements in the first paragraph.

**Comment**: Third paragraph, before objectives and perspectives, about the knowledge gaps on this topic and why it is important to study them: we need to estimate the burden of under-nutrition among people living with HIV in sub-Saharan Africa and understand what are the main risk factors for this population. If the focus in on adults among ART (which again is not clear in the title and abstract), this has to be better justified too.

**Response**: In the third paragraph, we presented a summary of previous key findings related to the topic and showed the evidence gap and how our study contributed to filling it. The reason for categorizing based on ART status is that there is evidence that indicates taking ART improves nutritional status, and sub-group analysis by ART status will enable us to appreciate the proportion separately.

**Methods**:

**Comment**- I don’t understand the comparison/control group here. Most studies selected don’t compare the prevalence of under-nutrition by HIV status, and the following results will not really display this. Does the comparative sample was really a criterion of the search strategy?

**Response**: In cross-sectional studies, the comparator group is identified by context or assumption. The results are interpreted under this assumption. However, in case-control and cohort studies, the control groups are those who are HIV-negative adults. The comparative sample was not a criterion of the search strategy but a context for interpretation.

**Comment**: Age threshold at age 16 years also need justification, why include youth for which nutritional and growth outcomes are still evolving greatly compared to adults?

**Response**: The lowest age categories for adults in the included studies were 15 years, 16 years, 18 years, and 20 years. Adults are defined as people over the age of "15 years" by the UNAIDS definition. It is corrected in the PICO. <https://www.unaids.org/en/resources/fact-sheet>

**Comment:** What was the period of time selected for the searching strategy? It is said until November 2021 but no information about the beginning, only in the title of the figures (2009), what is the justification for this threshold?

**Response:** Accepted. We include the commencement period. The reason was that there was a previous meta-analysis study on the prevalence of undernutrition among women living with HIV published in 2008. To avoid redundancy and add evidence to existing knowledge, we took published studies starting in 2009 and included both adult males and females. <https://bmcpublichealth.biomedcentral.com/articles/10.1186/1471-2458-8-226>

**Comment- I would suggest creating a table for the search strategy rather than putting it in the text, to better highlight it.**

**Response:** It is possible to present search strategy in table.

**Results: Need further details for the reader to understand and have the full picture**

**Comment-** Table 2: Results should be much more specific and includes for each selected article: gender/sex distribution, ART duration (at least +/- 6 months on ART), the type of setting (urban, semi-urban, rural), median age or age range if available, the associated factors measured (so we know for each pooled analysis how many articles you included). I don’t think the response rate is useful here. Add the prevalence with its 95% confidence interval or interquartile range (fig 3 seems to display it but is not easy to read and I would suggest to delete it).

**Response:** We include some of the suggested variables, like 95% CI and mean/median age, but inserting all variables will overwhelm the table and make it unattractive. Studies on ART duration were conducted in both categories, i.e., no study was conducted on either the -6 or +6 month separately. Most importantly, the data on the associated factors, including ART duration, are extracted on a separate Excel sheet. I will attach it as a supplementary file for further clarity. The response rate is added to show the credibility of the reported estimate. The forest plot for each associated factor would tell us the number of studies included there. For better understanding, we created a table that summarizes the points related to the associated factors (Table 4).

Figure 3 is a sensitivity analysis graph. It, like the funnel plot, serves as a visual guide to publication bias. The graph shows the effect estimates (indicated by small circles) are within the confidence interval limit, and no outlier is detected. Thus, the removal of studies to minimize publication bias is not required. The sensitivity analysis indirectly supports the risk of bias assessment, which was arbitrarily assessed using NOS. We believe adding this graph will enhance the quality of this manuscript.

**Comment: Fig5: can this figure be stratified also by duration on ART? (whether +/- 6 months, 1 year or 5 years, depending on your results)**

**Response:** No, it is not possible. In order to stratify by the duration of ART, individual studies should study patients with a specific category of treatment duration. Studies were not conducted with a certain limit on treatment duration. Most importantly, the pooled estimate of the adjusted odds ratio between "duration of ART" and "undernutrition" is presented in the "results" section and was 2.68.

**Comment:** Fig 6,7 and 8: it is not specific what was the reference group for each study. For example, for WHO clinical stage, does all the studies compared stage 3-4 with stage 1-2? What was the comparison groups for CD4 count used to calculate the odds ratio? Same question for age and sex, how were the estimates calculated? If the definition and comparison were heterogeneous between studies, I don’t see the point of doing a pooled analysis, this could be a narrative review only for this part, with a table explaining the detailed results.

**Response:** We appreciate this outlook. We did not combine heterogenous comparison groups. We include the reference group that the majority of the studies used. We exclude articles that report on a completely different reference group. This is the major problem that researchers face during meta-analysis of determinants (associated factors) with outcome variables. We used the reported adjusted odds ratio to determine the strength of the association. We prefer to present a short summary of the associated factors and comparison group using a table format. (Table 4).

**Discussion: overall this part needs to be reinforced and better structured**

**Comment**: 1st paragraph: should summarized the results of the review first, not giving general messages, better suitable for the introduction of the end of the conclusion.

**Response**: We generally agree with the comment. We also understand that the first paragraph in the discussion summarizes major concepts outlined in the introduction and provides a logical linkage of the results with the original question or objectives. We make amendments based on the above.

**Comment**: 2nd paragraph: I don’t understand why comparing the results with a population of pregnant women, this is a specific topic. Also, why discussing the effects of undernutrition on mortality here? This outcome was not taken into account in the review.

**Response:** The comment is fully accepted. This was added due to the scarcity of similar meta-analysis studies for comparison with our findings. However, rather than compare with a different population and outcome variable, we decided to remove the comparators and mention the shortage of evidence under the limitation of study section.

**Comment**: In 3rd paragraph, you are discussing and comparing results by sex but this information is not well highlighted in you own article. What was the pooled prevalence by sex in your review? The comparison with results found on children are not necessary here. This will be more relevant to compare with the estimates of under-nutrition among the general population, to see if people living with HIV are more affected by this, and how much more affected.

**Response**: We presented the sex, age, ART duration, WHO clinical stage, and CD4 count in the result section under the sub-heading “Factors associated with under-nutrition”. Describing the prevalence by subgroup is different from determining the strengths of association between the factors and outcome variable. The pooled adjusted odds ratio is separately estimated for each above-mentioned factor. For clarity we will attach the data extraction excel sheet for all factors as a supplementary file.

**Comment**: Ref28: why discussing of discrepancy with your study while this review measured food insecurity and not under-nutrition, which are, as you said yourself, two different markers?

**Response:** Again, we also removed this section. It was inserted as food insecurity is closely related and leads to under-nutrition in majority of the studies. Similarly, this was used due to shortage of meta-analysis studies exactly carried out on under-nutrition.

**Comment**: Further in the same paragraph, comparing with studies focused on overweight and obesity seems not relevant as well, unless you incorporate those results in your definition of the study outcomes and describe it.

**Response:** Again, this comment is accepted. It was used for the above similar reason.

**Comment**: You briefly described the difference of prevalence per year in your results, I wonder if it would not be worth it to see and discuss if the prevalence has evolved over time, or at least comparing 2009-2015 and 2015-2021 for example. There might have been very few improvements, which is a result in itself, worthwhile to highlight.

**Response:** We appreciate you suggesting this perspective for interpretation, and we will include it in the result and discussion.

**Comment:** Other limitations need to be highlighted: heterogeneity and lack of comparative group. You should also discuss the risk of publication bias and how you mitigated it.

**Response:** High heterogeneity is expected in the meta-analysis of proportions reported from different countries. The source of heterogeneity was explored by sub-group analysis but not identified. The inter-country difference in population characteristics is obvious. It is also mentioned in the document under the sub-heading of quality appraisal results as "this result should be interpreted cautiously." We will include it in the limitation section. Regarding the publication bias, studies look symmetrically distributed in the funnel plot (Figure 2) but not by the Eggers statistical test. The sensitivity analysis graph (Figure 3) did not show outlier studies to be removed from the analysis. Trim and fill analysis is also another method, but it is not recommended as it provides artificial estimates. Additionally, the small study size effect is not the problem of this study, as 44 articles have been included in the analysis. Therefore, it is acceptable to run the analysis based on available studies, and publication bias will not be the limitation of this study.

**Response to reviewer #5**

**General comments:** Few areas in the manuscript require proof reading. Correct the sentence on page 4, paragraph 1, " For example, a study......times higher odds.............."

**Response:** Okay, accepted.

**Comment**: If possible, remove table 1 and you can just define the PICO questions by text. For this case, P can stand for population, I intervention and C: Comparison..... Also correct the sentence under population to read 16 years instead of 16 year... Also be consistent, is it grey literature or gray literature?

**Response**: Accepted. PICO questions can be formatted by texts using bullets. Though the words “grey” and “gray” can be used interchangeably we will use one of the terms throughout the paper.

**Comment**: Under Effect measures: Make sure you write that sentence in past tense "We included......"

**Response**: Accepted.

**Comment**: Methodology: Properly explain why I2 is high.

**Response:** Still, the source is not identified, but high heterogeneity is expected when combining proportions reported from different countries. The only thing we can do is add the statement "The result should be interpreted cautiously" and mention high heterogeneity under the limitation of the study section.

**Comment**: Discussion: Page 13, correct those 2 sentences " Conversely.........." " The study used....

**Response**: Okay, Accepted.

**Comment**: The second paragraph towards the end, you are discussing about children. This article is on adults.

**Response:** The paragraph on children was added due to scarcity of available similar studies on adults. However, we delete it as it is beyond the scope of our study defined in the PICO.
